# Health-economic outcomes in hospital patients with medical-psychiatric comorbidity: A systematic review and meta-analysis

**DOI:** 10.1371/journal.pone.0194029

**Published:** 2018-03-13

**Authors:** Luc Jansen, Maarten van Schijndel, Jeroen van Waarde, Jan van Busschbach

**Affiliations:** 1 Erasmus MC, University Medical Center Rotterdam, Department of Psychiatry, Rotterdam, the Netherlands; 2 Zilveren Kruis Achmea, Department of Health Procurement, Leusden, the Netherlands; 3 Rijnstate Hospital, Department of Psychiatry, Arnhem, the Netherlands; Weill Cornell Medicine-Qatar, QATAR

## Abstract

**Background:**

Hospital inpatients often experience medical and psychiatric problems simultaneously. Although this implies a certain relationship between healthcare utilization and costs, this relationship has never been systematically reviewed.

**Objective:**

The objective is to examine the extent to which medical-psychiatric comorbidities relate to health-economic outcomes in general and in different subgroups. If the relationship is significant, this would give additional reasons to facilitate the search for targeted and effective treatments for this complex population.

**Method:**

A systematic review in Embase, Medline, Psycinfo, Cochrane, Web of Science and Google Scholar was performed up to August 2016 and included cross-references from included studies. Only peer-reviewed empirical studies examining the impact of inpatient medical-psychiatric comorbidities on three health-economic outcomes (length of stay (LOS), medical costs and rehospitalizations) were included. Study design was not an exclusion criterion, there were no restrictions on publication dates and patients included had to be over 18 years. The examined populations consisted of inpatients with medical-psychiatric comorbidities and controls. The controls were inpatients without a comorbid medical or psychiatric disorder. Non-English studies were excluded.

**Results:**

From electronic literature databases, 3165 extracted articles were scrutinized on the basis of title and abstract. This resulted in a full-text review of 86 articles: 52 unique studies were included. The review showed that the presence of medical-psychiatric comorbidity was related to increased LOS, higher medical costs and more rehospitalizations. The meta-analysis revealed that patients with comorbid depression had an increased mean LOS of 4.38 days compared to patients without comorbidity (95% CI: 3.07 to 5.68, I2 = 31%).

**Conclusions:**

Medical-psychiatric comorbidity is related to increased LOS, medical costs and rehospitalization; this is also shown for specific subgroups. This study had some limitations; namely, that the studies were very heterogenetic and, in some cases, of poor quality in terms of risk of bias. Nevertheless, the findings remain valid and justify the search for targeted and effective interventions for this complex population.

## Introduction

Hospital inpatients often experience medical and psychiatric problems simultaneously. For patients who are admitted to hospitals for a general medical illness, the prevalence of a comorbid psychiatric disorder is estimated at 40% [1]. Conversely, psychiatric patients are at increased risk of developing comorbid general medical disorders [2]. The consequences of concurrent general medical and psychiatric illnesses include an increase in morbidity, mortality, healthcare utilization and costs [3]. All psychiatric disorders are associated with increased morbidity, and mortality and patients who suffer from eating disorders and substance dependence have the highest risk[4–7]. Numerous studies examined the relationship between medical-psychiatric comorbidities and health-economic outcomes. Most studies have evaluated the impact of comorbidities on three outcome measures: length of stay (LOS), medical costs and rehospitalization rates [8–12]. These parameters are considered major cost drivers in healthcare [13]. Although these studies suggest that medical-psychiatric comorbidities relate to health-economic outcomes, these outcomes have never been reviewed systematically. Furthermore, the nature of the relationship may be different for separate subgroups [8, 11, 14, 15]. Meta-analyses on this subject have not been performed and, therefore, the overall pooled effect of the relationship between medical-psychiatric comorbidities and health-economic outcomes remains unknown.

### Aims of the study

The aims of our study were as follows: 1) a systematic review of the literature to examine the relationship between medical-psychiatric comorbidity and health-economic outcomes; 2) an examination of possible differences in health-economic outcomes in patient subgroups; 3) an examination of the pooled-effect sizes for the effects of medical-psychiatric comorbidities on LOS, medical costs and rehospitalizations using meta-analyses. The importance of this review lies in its contribution to a cost-effective treatment of this complex and expensive population [16]. Policy makers might use the estimation of pooled-effect sizes for the effects of medical-psychiatric comorbidity on the examined health-economic outcomes to improve the delivery of cost-effective care for patients with medical-psychiatric comorbidities. Furthermore, this review might stimulate future researchers to examine the impact of several subgroups with comorbidity on different health-economic outcomes more thoroughly.

## Method

The literature was scrutinized by making queries in electronic literature databases and by examining cross-references in the included articles. Six electronic literature databases were examined: Embase, Medline, Cochrane, Psycinfo, Web of Science and Google Scholar. The data extraction was performed on March 3, 2015 by a biomedical information specialist and updated on August 1, 2016: the search terms used are shown in Table 1 and an example of a full electronic search is provided in Table 2. The search terms were adjusted to comply with the specifications of the different databases. Duplicates and non-English articles were removed. A literature search based on cross-referencing was performed on November, 27, 2015. As no new articles were included after updating the search on August 1, 2016, no new cross-referencing was performed after this point.

**Table 1:** Search terms systematic review.

psychosomatics OR somatic* OR physical* OR medical* OR medicine NEAR psychiatr* OR mental* OR cognit* OR psychosomatic* AND comorbidity OR comorbid* multi* OR poly OR co NEAR morbid* OR patholog*OR co OR coexist* OR disorder* AND 'hospital patient OR hospitalization OR outpatient OR 'outpatient care' OR 'ambulatory care OR 'outpatient department OR hospital OR general hospital OR hospital admission OR 'hospital care OR 'university hospital OR 'hospital discharge OR hospital department OR ward OR hospital* OR inpatient* OR outpatient* OR ward* OR ambulatory*).

**Table 2:** Full electronic search strategy in Embase.

(psychosomatics/exp OR (((somatic* OR physical* OR medical* OR medicine) NEAR/6 (psychiatr* OR mental* OR cognit*)) OR psychosomatic*):ab,ti) AND (comorbidity/exp OR 'cluster analysis'/exp OR (comorbid* OR cluster* OR ((multi* OR poly OR co) NEAR/3 (morbid* OR patholog*)) OR (co NEXT/1 exist*) OR coexist* OR (mixed NEAR/3 disorder*)):ab,ti) AND ('hospital patient'/exp OR hospitalization/exp OR outpatient/exp OR 'outpatient care'/exp OR 'ambulatory care'/exp OR 'outpatient department'/exp OR hospital/de OR 'general hospital'/de OR 'hospital admission'/exp OR 'hospital care'/exp OR 'university hospital'/exp OR 'hospital discharge'/exp OR 'hospital department'/de OR ward/de OR (hospital* OR inpatient* OR outpatient* OR ward* OR ambulator*):ab,ti) NOT ([Conference Abstract]/lim OR [Letter]/lim OR [Note]/lim OR [Conference Paper]/lim OR [Editorial]/lim) AND [english]/lim

### Inclusion criteria

Empirical studies that examine the effect of having either medical or psychiatric comorbidity in hospital inpatients are included. These hospital inpatients have to be either primarily medically or psychiatrically ill and aged 18 years or older. Studies had to measure the effect on any of three major health economic outcomes (length of stay and/or medical costs and/or rehospitalization), and must be published in a peer-reviewed journal. Any measure of LOS (days, mean, median, et cetera), all types of medical costs (direct and indirect), and all reports of rehospitalization (all relevant time-frames) were included as the aim was to broadly review the literature on these outcomes. Inclusion criteria were limited to these outcomes since these are most reported in literature. It was hypothesized that medical-psychiatric comorbidity has a significant impact on these outcomes; it was further expected that effects were most prominent for inpatients, because these patients are presumed to be the most severely ill. By focusing on these inclusion criteria, we aimed to decrease heterogeneity between included studies. Consequently, study design was not an exclusion criterion, there were no restrictions on publication dates. Additionally, the examined populations had to consist of inpatients with medical-psychiatric comorbidities and controls. The controls were required to be patients without a comorbid medical or psychiatric disorder; however, the way the controls were sampled was not an exclusion criterion. Non-English articles were excluded.

Ideally, a comparison of comorbid patients (disease A and B) with patients that only have disease A or only disease B would be made. In this way, discerning whether the effect was additive or multiplicative could be estimated. However, only inpatients with disease A or B were included and compared with disease AB for reasons of feasibility.

### Selection procedure

Two authors (LAWJ and MAvS) independently assessed 100 randomly selected titles and abstracts to validate the inclusion criteria. All obtained articles were then reviewed on the basis of title and abstract by the first author. Full texts of the included articles were then obtained. Subsequently, two authors (LAWJ and JAvW) independently assessed the selected articles in a standardized manner to further include or exclude articles for the review. Consensus was sought when disagreements between the authors on inclusion existed. When no consensus could be reached, the assessment of a third author (MAvS) was decisive. An intra-class correlation coefficient was calculated to examine the accuracy of match between the reviewers.

Thereafter, two authors (LAWJ and JAvW) read all included articles and extracted relevant data about nine predetermined characteristics: study design, patient characteristics, somatic diagnoses, psychiatric diagnoses, control group, moment of data collection (during or after treatment), LOS, medical costs, and rehospitalization rates. An electronic spreadsheet (Microsoft Excel) was used with these predetermined characteristics and available information for every included article was recorded. Again, disagreements on the collected data were resolved by discussion and, if no consensus was reached, the assessment of a third author (MAvS) was decisive.

The Newcastle-Ottawa Scale (NOS) for assessing the quality of non-randomized studies in meta-analyses was used to assess the risk of bias in each study [17]. The NOS scale has been developed to assess the quality of non-randomized studies with its design. It uses a “star system” that judges a study on three broad perspectives: selection of groups, comparability of groups, discernment of either the exposure or outcome of interest for case-control or cohort.

All included studies were rated by the first author (LAWJ) based on the NOS; using NOS, each article was rated on nine variables and could earn a maximum of “9 stars”. More stars indicates less risk of bias in the assessed article. Finally, in reporting this review, the “Preferred reporting Items for Systematic reviews and Meta-Analyses” (PRISMA) checklist [18] was used. A review protocol was not used a priori to this systematic review.

Subgroups were selected based on the number of studies that researched the specific subgroup. Only the most extensive researched subgroups per health-economic outcome are presented in this study.

### Meta-analyses

Meta- analyses were performed using Review Manager 5.3. In studies that reported continuous data, only those that stated means and standard deviations were included in the analyses. Furthermore, pooled-effect sizes were only reported if the (sub-)population was reasonable homogenous as this is not appropriate for heterogeneous studies [19]. The heterogeneity was based on statistics where a cut-off point of an I^2^ of 50% was used. A random-effects model was applied to calculate treatment effects. In order to express treatment effects, the (standard) mean differences and 95% confidence intervals (CI) were calculated. The X^2^ test was used to determine the heterogeneity between included studies where I^2^ values of <25% represent low heterogeneity, between 25% and 50% represent moderate heterogeneity and values >50% suggest severe heterogeneity between the studies [20]. Statistical significance was assumed at *P* <0.05.

## Results

### Study selection

**Fig 1:**
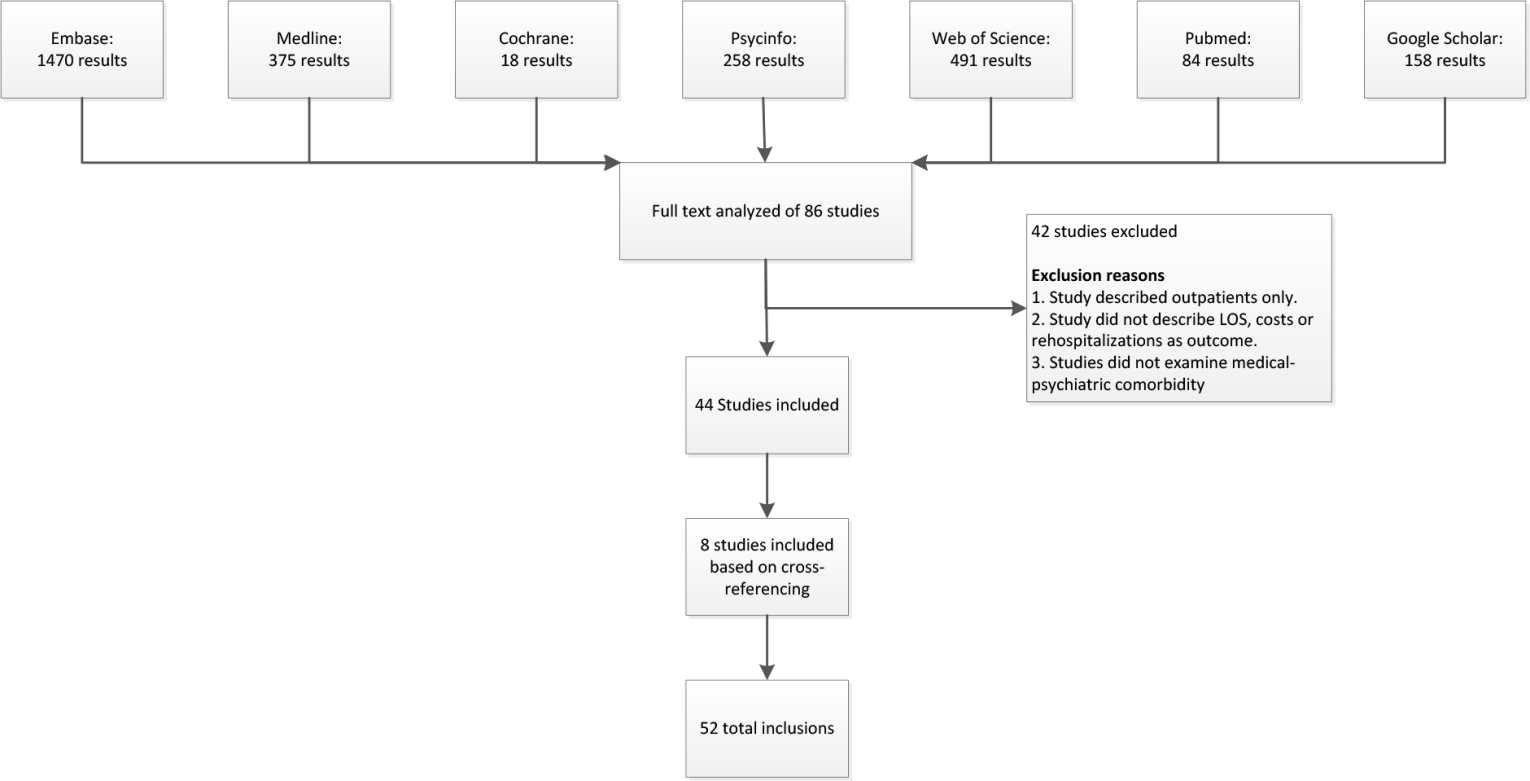
Flowchart of study results.

The systematic literature search resulted in a total of 6163 articles. After removing duplicates and non-English articles, a total of 3165 studies remained. After reviewing all studies on title and abstract, a total of 86 articles were included for the full-text review. The reviewing authors agreed on the inclusion of 36 articles and the exclusion of 37 articles; an intra-correlation coefficient of 0.76 between the reviewers was estimated. Consensus was not reached for thirteen articles; after discussion, eight of these were included and five excluded. References in all 44 included articles were cross-checked. This resulted in ten extra articles to review in full text, eight of which were included. These studies did not appear in the search since the title did not refer to medical-psychiatric comorbidity but to a specific disorder. Finally, after the selection procedure, a total of 52 articles entered the definite literature review (“Fig 1”).

A variety of study designs were included, mostly observational in nature. Longitudinal cohort study was the most common design. No randomized clinical trials were found.

All studies compared either a medical or psychiatric index disorder, 48 articles examined patients with a medical index disorder and a psychiatric comorbid disorder and, conversely, four articles examined atiens with a psychiatric index disorder and a medical comorbid disorder. The control groups consisted of inpatients without a medical or psychiatric comorbid disorder. The number of participants in the reviewed studies ranged from 63 to 1,617,710 patients. The impact of medical-psychiatric comorbidities on LOS was described in 42 (81%) articles, the impact on costs in 12 (23%) articles and on rehospitalization in ten (19%) articles. The risk of bias was assessed using the NOS and the scores ranged from the minimum (one star) to the maximum number (nine) of stars. Details of the individual studies are presented in Table 3, which is divided by outcome measure and sorted according to the NOS grading system. No additional risk of bias was performed: the risk of publication bias was low since both negative and positive findings were of interest for all measures (LOS, costs, rehospitalization).

**Table 3:** Impact of medical-psychiatric comorbidity on length of stay (LOS), medical cost and rehospitalization.

**Impact of medical psychiatric comorbidity on Length Of Stay**						
**Study**	**N**	**Index disorder**	**Comorbid disorder**	**Control group**	**Length-of-stay (LOS) in days**	**NOS**
Mai et al. [21], 2011	433.388	Diabetes, COPD, congestive heart failure, convulsions and epilepsy.	Alcohol/drug disorders, Schizophrenia Affective psychoses, Other psychoses Neurotic disorders, Personality disorders Adjustment disorders, Depressive disorders, Other mental disorders	No psychiatric comorbidity	Average LOS with comorbidity 6.1 vs. without comorbidity 4.4.	9
Furlanetto et al.[22], 2003	317	Cardiovascular, Gastrointestinal, Neoplasms, Pulmonary, Infectious	Cognitive impairment, depressive disorders, substance related disorders, adjustment disorders, anxiety disorders	No psychiatric comorbidity	Mean LOS with comorbidity 14.7(SD 13.8) vs. without comorbidity 12.1(SD 9.9), LOS with comorbid cognitive impairment significantly prolonged (F = 17.8 P<0.01).	8
Bressi et al. [23], 2006	1.617.710	In order to identify patients hospitalized for medical conditions, patients having a primary mental diagnosis were excluded	Schizophrenia, major mood disorders and substance abuse disorders	No psychiatric comorbidity	Mean LOS with comorbidity 0.15 days longer (P<0.001) vs. patients without comorbidity. Mean LOS with comorbid schizophrenia 0.86 days longer (P<0.0001) Mean LOS with comorbid mood disorder 0.26 days longer (P<0.0001) Mean LOS with substance abuse 0.25 shorter (P<0.001)	8
Hansen et al. [24], 2001	157	Consecutive inpatients department of internal medicine	Patients with comorbid mental illness	No psychiatric comorbidity	OR LOS> = 10 days with any mental disorder = 0.5 (0.2–1.3) OR LOS> = 5 days with any mental disorder = 0.9 (0.4–2.4)	8
Saravay et al. [25], 1991	278	Patients from medical, surgical and gynecology floor	Psychiatric illness; measured with MMSE, Zung Depression Inventory, SCL-90	No psychiatric comorbidity	Mean LOS is significantly related with comorbid organicity (p = 0.004), depression (p = 0.03), and anxiety (p = 0.05)	8
Bourgeois et al. [26], 2005	31.846	All medical diagnosis	All psychiatric illness	No psychiatric comorbidity	Mean LOS with comorbid adjustment disorder 5.68 (1999), 7.96 (2000) 8.85 (2001) vs. no psychiatric disorder and substance use disorders 3.29, 3.43, 3.51 respectively (P<0.001).	8
Benzer et al. [27], 2012	21.716	All medical diagnosis	Patients with post discharge mental health care	No psychiatric comorbidity	Mean LOS with post-discharge mental health care: 7.86 (SD21.1) vs no mental health care post-discharge: 7.2 (SD15.4) days (non sig.)	8
Fulop et al. [28], 1989	66.637	Craniotomy, nervous system neoplasm, cerebrovascular disorder, respiratory neoplasm, bronchitis and asthma, circulatory disorder, heart failure l, bowel procedure, digestive malignancy, cirrhosis, renal failure, chemotherapy, operation room procedure	All psychiatric diagnosis	No psychiatric comorbidity	All 13 somatic diagnosis related groups (DRG) with psychiatric comorbidity have significant longer mean LOS than without comorbidity.	8
Levenson et al. [29], 1990	455	All medical diagnosis	Very depressed; very anxious; cognitive impairment; high pain levels;	Low level of psychopathology	Mean LOS in high level patients: 11.5 (SD12.4) in low level 8.7 (SD11.9) (P<0.001)	8
Fulop et al. [30], 1987	59.259	All medical and surgical patients	Organic mental disorder (delirium, substance abuse)	No psychiatric comorbidity	Mean LOS with comorbidity: 19.8 (SD33.3) vs. without 9.2 (SD15.3) (p– 0.001) in NYC; 13.7 (SD27.7) vs. 8.3 (SD13.2) in Chicago (p– 0.001)	7
Hochlehnert et al. [11], 2011	1063	Cardiovascular inpatients	Depressive disorders, anxiety disorders, somatoform disorders, organic psychiatric disorders, adjustment disorders, substance dependence, schizophrenic disorders, and other diagnosis	No psychiatric comorbidity	Mean LOS of patients with psychiatric comorbidity significantly longer compared to patients without psychiatric comorbidity (F1.11 = 34.04; p<0.001)	7
Schubert et al. [31], 1995	532	Psychosis, depression, personality disorder, anxiety disorder, adjustment disorder, bipolar disorder, other psychiatric disorders	Physical illness	No somatic comorbidity	Mean LOS significant longer with comorbidity 19.31 vs. without 13.13. Depression with somatic comorbidity significant longer 20.08 (SD 24.8) than without 11.48 (SD 11.88)	7
Zatzick et al. [32], 2000	10.561	Diabetes, hypertension, chronic liver disease, ischemic heart disease, degenerative nervous conditions, epilepsy, obesity, and coagulation defects, HIV infection	Alcohol abuse, alcohol dependence, drug abuse, drug dependence, anxiety disorders, bipolar disorders, childhood disorders, delirium, dementia, depression, disorders attributable to organic brain damage, personality disorders, psychoses, stress disorders, and other disorders	No psychiatric comorbidity	Mean LOS 10% shorter with alcohol abuse (p = <0.01) than without. Mean LOS 60% to 103% longer with delirium, psychoses and stress-disorder (p = <0.01) than without.	7
Koenig et al. [33], 1998	542	60 years and older cardiology and neurology patients	Depressed patients;	No psychiatric comorbidity	Mean LOS with comorbid major depression 12.1 (SD19.8) vs. without depression 5.7 (SD12.8) (p = <0.001)	7
Fulop et al. [34], 1998	467	Patients 65 years or older with a medical disorder	Cognitive impairment, depression or anxiety disorder	No psychiatric comorbidity	Mean LOS with comorbidity 13.1 (SD13.0) vs. without 10.5 (SD11.7) (P = 0.025). LOS with depressive disorder 11.0 (SD13.1) vs. without 11.8 (SD12.5) (P = 0.51)	7
Adams et al. [8], 2015	12.283	Patients 65 years or older with a medical disorder	Organic, substance abuse, schizophrenia, mood, neurotic/stress, physiological/physical, personality disorder	No psychiatric comorbidity	Mean LOS with comorbidity 16.06 vs. without 11.5 (p = <0.001)	7
Ismail et al. [12], 2014	477	All medical diagnosis	Dementia; subgroup psychosis (with or without dementia)	No psychiatric comorbidity	Mean LOS geriatric dementia patients 74.7 (SD93.7) without dementia 69.9 (SD87.5). Geometric mean LOS 38.1 with dementia vs. 34.6 without (p = 0.32)	7
Hosaka et al. [35], 1999	65	Malignancy	Major depression	No psychiatric comorbidity	Mean LOS benign with major depression 135.0 (SD160.7) without 69.7(SD61.9) (P = <0.05).	7
Sayers et al. [36], 2007	20.429	Patients 65 years of older with one acute care hospitalization of congestive heart failure.	Alcohol abuse, drug abuse, psychosis, depression, bipolar disorders, anxiety disorders, and other psychiatric conditions	No psychiatric comorbidity	Comorbid psychoses additional mean LOS 1.06 days (P = <0.001) Comorbid depression additional mean LOS 0.89 days (P = <0.001) Comorbid bipolar disorder additional mean LOS 1.43 days (P = 0.02)	7
Smith et al. [37], 2014	63	Idiopathic Pulmonary Fibrosis (IPF), COPD, CF	Delirium	No psychiatric comorbidity	Presence of delirium was associated with longer duration of hospitalization (p = 0.006)	6
Wancata et al. [38] 2001	821	Diseases of the circulatory system, diseases of digestive and genitourinary system	Dementia, minor depression and substance abuse disorders	No psychiatric comorbidity	Mean LOS with comorbidity 17.6 vs. without 11.5. Dementia 1.35(1.16–1.57) substance abuse disorders 1.24(1.04–1.48) Alcohol- & drug related psychiatric disorders 1.54(1.13–2.11) significantly associated with longer LOS	6
Ceilley et al [39]., 2005	87	Depressive disorder, bipolar disorders, psychotic disorders	Osteoarthritis, viral hepatitis C COPD	No somatic comorbidity	Mean LOS with somatic comorbidity 12.3 (SD5.2) vs. 9.1 (SD 3.7) (P = 0.003)	6
Davydow et al. [40], 2011	3.591	Diabetes	Depression	No psychiatric comorbidity	Mean LOS no depression 7.9 (SD9.9) major depression 12.2 (SD16.8) (P<0.001)	6
Chwastiak et al. [9], 2014	82.060	Diabetes, Heart failure, renal failure, hypertension complicated, peripheral vascular	Bipolar disorder, schizophrenia, psychotic disorders delusional disorder and nonorganic psychoses.	No psychiatric comorbidity	No comorbidity median 3 days Inter Quartile Rang (IQR): 2–4 vs. severe Mental illness: median 3 days IQR: 2–4	6
Bourgeois et al. [41], 2006	155	All medical diagnosis	Delirium, dementia, and both	General hosp. population	Mean LOS with comorbidity 13 vs. 3 without.	6
Bourgeois et al. [15], 2009	157	Hospital population	Cognitive disorders (primarily dementia and delirium or both)	No psychiatric comorbidity	Mean LOS with comorbidity 18.6 vs. 3 without.	6
Stevens et al. [42], 1998	42	All medical diagnosis	Delirious patients	No psychiatric comorbidity	Median LOS cases 20.0 (1–117) vs. controls 8.0 (1–171), delirious LOS sig. longer 2.2 (1.5–3.3) than controls	6
Uldall et al. [43], 1994	357	Aids	Mood disorders, substance use disorders, organic psychiatric disorders, anxiety disorders, and adjustment disorders	No psychiatric comorbidity	LOS with comorbidity 16.8 (SD15.0) vs. without 10.2 (SD19.1) (P = 0.01)	6
Borckardt et al. [44] 2011	10.865	All medical diagnosis except emergency room stays	Patients receiving outpatient treatment	No psychiatric comorbidity	Mean LOS with inpatient psychiatry consultation 9.39 vs. without 4.63 (P = <0.001)	6
Boustani et al. [45], 2010	995	Patients 65 or older admitted to medical services	Delirium	No psychiatric comorbidity	Mean LOS with comorbid delirium 9.2 vs. 5.9 without (P = <0.001)	5
Morris et al. [46], 1990	110	Peptic ulcer parenchymal liver disease intestinal malignancy	General Health Questionnaire (GHQ) case	GHQ noncase	Mean LOS with comorbidity 8.7 (SD5.9) vs. without 8.3 (SD5.7) (non-significant)	5
Verbosky et al. [47], 1993	48	All	Depression	No psychiatric comorbidity	Mean LOS with comorbid depression 20 (range 2–95) vs. without 10 (range 2–51) (P = 0.02)	5
Erdur et al. [48], 2012	41	Anorexia Nervosa (AN)	Predominantly internal diseases	No somatic comorbidity	Mean LOS with somatic comorbidity 66.6 (SD50.3) vs. without 50.0 (SD47.0) (P = 0.05)	4
Sloan et al. [49], 1999	2323	Psychosis, depression, personality disorder, anxiety disorder, adjustment disorder, bipolar disorder, other disorders	Physical illnesses were limited to those appearing in the (ICD 9)	No somatic comorbidity	Mean LOS with somatic comorbidity 20.0 vs. without 16.6 (P = <0.001)	4
Ackerman et al. [50], 1988	92	All medical diagnosis	A form of depressive disorder	No psychiatric comorbidity	Mean LOS with comorbid depression 2.52 days longer than without (P<0.001)	4
Uldall et al. [51], 1998	2834	AIDS	Dementia, delirium, schizophrenia, psychosis, depression, bipolar-, anxiety-, adjustment-, personality-disorder, alcohol-, drug-dependence, alcohol-, drug-abuse	No psychiatric comorbidity	Median LOS with comorbidity 9.0 vs. without 7.0 (P = <0.001)	4
McCusker et al. [52], 2003	359	Patients 65 or older with a medical admission	Delirium	No psychiatric comorbidity	Mean LOS prevalent delirium 16.2 (SD13.2) vs. without 12.6(SD11.8) (non sig.) Mean LOS incident delirium 20.2 (SD14.2) vs. without 10.7(SD9.8) (sig.)	4
Creed et al. [53], 2002	263	Patients admitted to an acute medical ward	Depression and anxiety	No psychiatric comorbidity	Median LOS with comorbidity 9.0 (6–20) vs. 7.0 (4–18) without (non sig.)	4
Schubert et al. [54], 1992	31	Stroke or Amputation	Depression, according to the geriatric depression scale	No psychiatric comorbidity	Correlation CVA and depression +0.575 (P<0.05) an between amputation patients and depression +0.266 (non. sig) indicating longer LOS	4
Douzenis et al. [55] 2012	428	Schizophrenia and bipolar patients	Endocrine Circulatory Nervous Respiratory Musculoskeletal Blood Skin	No somatic comorbidity	Mean LOS with comorbid bipolar disorder 16.8 (SD8.8) was significantly lower than comorbid schizophrenia 19.57 (SD11.2).	4
Mojet et al. [57], 1989	17687	All medical diagnosis	Consultation Liaison (C-L)	No CL consultation	Mean LOS with CL 26.1 vs. without 11.1	4
Johansen et al. [56] 2012	*Not reported	Patients admitted to an acute care medical ward	Patients with mental illness (most: organic (delirium/dementia), mood disorders and schizophrenia)	No psychiatric comorbidity	Average LOS with comorbidity 15.3 vs. without 5.8. Comorbidity 2.7-fold increase in LOS vs. without comorbidity.	1
**Impact of medical psychiatric comorbidity on medical costs**						
**Study**	**N**	**Index disorder**	**Comorbid disorder**	**Control group**	**Length-of-stay (LOS) in days**	**NOS**
Benzer et al. [27], 2012	21.716	All medical diagnosis	Patients with post discharge mental health care	No psychiatric comorbidity	Total cost (inpatient, outpatient and pharmacy costs) ($) with mental health care post-discharge: 29.566 (SD 31.577) vs. without 20.611 (SD 26.855) (non sig.)	8
Levenson et al. [29], 1990	455	All medical diagnosis	Very depressed; very anxious; cognitive impairment; high pain levels;	Low level of psychopathology	Mean total hospital costs high level patients 7634 (SD10484) dollar vs. low level 5643 (SD7411) dollar (P = <0.003)	8
Hochlehnert et al. [11]^,^ 2011	1063	Cardiovascular inpatients	Depressive disorders, anxiety disorders, somatoform disorders, organic psychiatric disorders, adjustment disorders, substance dependence, schizophrenic disorders, and other diagnosis	No psychiatric comorbidity	Average total cost with psychiatric comorbidity 7663 (SE571) vs. without 5142 (SE210) (sig.)	7
Druss et al. [58], 1999	77.183	All medical diagnosis	Major depression, depressive symptoms only, substance abuse, comorbid depression and substance abuse	No psychiatric comorbidity	Total increased inpatient costs compared to patients without these comorbidities: depression/substance abuse 1033$, depressive symptoms 861$, major depression 1581$, substance abuse 1244$, depression with substance abuse 4681$ (P<0.001)	7
Haas et al. [10], 2012	127	Anorexia Nervosa	All medical diagnosis	No anorexia nervosa	Number of comorbidity groups per patient is not significantly related to increased costs gamma -0.018(0.02)	7
Zatzick et al. [32], 2000	10.561	Diabetes, hypertension, chronic liver disease, ischemic heart disease, degenerative nervous conditions, epilepsy, obesity, and coagulation defects, HIV infection	Alcohol abuse, alcohol dependence, drug abuse, drug dependence, anxiety disorders, bipolar disorders, childhood disorders, delirium, dementia, depression, disorders attributable to organic brain damage, personality disorders, psychoses, stress disorders, and other disorders	No psychiatric comorbidity	Costs 10% decrease with alcohol abuse (p = <0.01) than without. Costs 60% to 103% increase with delirium, psychoses and stress-disorder (p = <0.01) vs. without. Total costs in patients with delirium, psychoses, and stress-disorders 46% to 93% higher costs vs. no comorbidity.	7
Adams et al. [8], 2015	12.283	Patients 65 years or older with a medical disorder	Organic, substance abuse, schizophrenia, mood, neurotic/stress, physiological/physical, personality disorder	No psychiatric comorbidity	Hospital costs with mental illness $24.076 (SD49.320) vs without mental illness $10.473 (SD17.391) (P = < 0.001)	7
Sayers et al. [36], 2007	20.429	Patients 65 years of older with one acute care hospitalization of congestive heart failure.	Alcohol abuse, drug abuse, psychosis, depression, bipolar disorders, anxiety disorders, and other psychiatric conditions	No psychiatric comorbidity	Psychiatric comorbidities, associated with higher total hospitalization costs 7.294$ (P = 0.001).	7
Shen et al. [59], 2008	2440	Asthma, diabetes, heart disease hypertension and osteoarthritis	Affective disorders, anxiety, somatoform, dissociative, personality disorders; schizophrenia	No psychiatric comorbidity	Mean inpatient costs with mental illness 2.731$ vs. without mental illness 2072$ (non sig.)	6
Borckardt et al. [44], 2011	10.865	All medical diagnosis except emergency room stays	Patients receiving outpatient treatment	No psychiatric comorbidity	Mean total costs patients receiving psychiatry consultation 25.773$ vs. without consultation 9672$ (p < .001)	6
Welch et al. [60], 2009	618.780	Asthma, back pain, diabetes, epilepsy, headache, hypertension, IVDD, obesity, joint pain, CHF, CAD	Depressed	No psychiatric comorbidity	Inpatient costs significantly increased in coronary artery disease 1890$, epilepsy 2.560$ and congestive heart failure 13900$ vs. no comorbid depression	5
Creed et al. [53], 2002	263	Patients admitted to an acute medical ward	Depression and anxiety	No psychiatric comorbidity	Mean total healthcare costs cases $8,541 (SE $605) vs. without $5,857 (SE $859) (P = 0.01)	4
**Impact of medical psychiatric comorbidity on rehospitalization**						
**Study**	**N**	**Index disorder**	**Comorbid disorder**	**Control group**	**Length-of-stay (LOS) in days**	**NOS**
Kartha et al. [61], 2007	144	Medical inpatients	Major depression	non-rehospitalisation	Comorbid depression tripled the odds of rehospitalization (OR = 3.3) (95%CI = 1.2 to 9.3)	8
Saravay et al. [62], 1996	273	Medical and surgical inpatients	Depression, obsessive compulsive-, anxiety disorder, psychoticism, hostility, interpersonal sensitivity.	No psychiatric comorbidity	Compared to the rest of the study group, the cognitively impaired patients averaged twice as many rehospitalizations (sig.)	8
Adams et al. [8], 2015	12.283	Patients 65 years or older with a medical disorder	Organic, substance abuse, schizophrenia, mood, neurotic/stress, physiological/physical, personality disorder	No psychiatric comorbidity	Rate of readmission in elderly with mental illness 1.87 (SD = 1.20) vs without 1.50 (SD = 1.03) (P < 0.001)	7
Chang et al. [63], 2001	164	Digestive and cardiovascular disease	Major depression and anxiety disorders	No readmission	No significant difference in readmission between patients with medical-psychiatric comorbidity and without	6
Chwastiak et al. [9], 2014	82.060	Diabetes, Heart failure, renal failure, hypertension complicated, peripheral vascular	Bipolar disorder, schizophrenia, psychotic disorders delusional disorder and nonorganic psychoses.	No psychiatric comorbidity	Increased odds of rehospitalization in patients with Serous Mental Illness vs. no SMI within next month (OR 1.24) (1.07–1.44) (p = 0.006)	6
Jiang et al. [64], 2001	374	Congestive heart failure	Mild or major depression	No psychiatric comorbidity	Major depression associated with increased odds of readmission at 3months (OR, 1.9 P = 0.04) and one year (OR = 3.07 P = 0.005)	6
Borckardt et al. [44], 2011	10.865	All medical diagnosis except emergency room stays	Patients receiving outpatient treatment	No psychiatric comorbidity	Number of hospitalizations within 6 months with psychiatric comorbidity 1.6 vs. without 1.34 (p = .001)	6
Boustani et al. [45], 2010	995	Patients 65 or older admitted to medical services	Delirium	No psychiatric comorbidity	Readmission within 30 days after discharge with delirium 22.5% vs. without 17.8% (P = 0.50)	5
Uldall et al. [51], 1998	2834	AIDS	Dementia, delirium, schizophrenia, psychosis, depression, bipolar-, anxiety-, adjustment-, personality-disorder, alcohol-, drug-dependence, alcohol-, drug-abuse	No psychiatric comorbidity	Median number of admissions with comorbidity 2 vs. without 1 (P<0.001)	4
Evans et al. [65], 1988	532	Medical/surgical patients	Psychiatric comorbidity was defined as any of the ICD-9-CM/DSM-3 psychiatric diagnosis codes	No psychiatric comorbidity	No significant difference in readmission rate between patients with mental disorders and without.	4

(*) Only the number of inhospital records is reported, not the number of patients.

### Results of individual studies

#### Impact of medical-psychiatric comorbidities on LOS

Table 3 shows the results of the 42 included studies that examined the impact of medical-psychiatric comorbidities on LOS. Articles with the least risk of bias are shown at the top of the table and are arranged according to the NOS grading system [8, 9, 11, 12, 15, 17, 21–57]. LOS was increased in patients with medical-psychiatric comorbidities compared to patients without comorbidity in 40 out of 42 (95%) articles; in 33 (79%) studies, this relationship was statistically significant [8, 11, 15, 23, 25, 26, 28–37, 39–45, 47–52, 55–57]. In five (12%) articles, this relationship was non-significant [9, 12, 27, 46, 53] and in four (10%) articles, no information about statistical significance was given [21, 22, 24, 38]. The remaining two (5%) studies did not show a relationship [9, 24].

**Fig 2:**
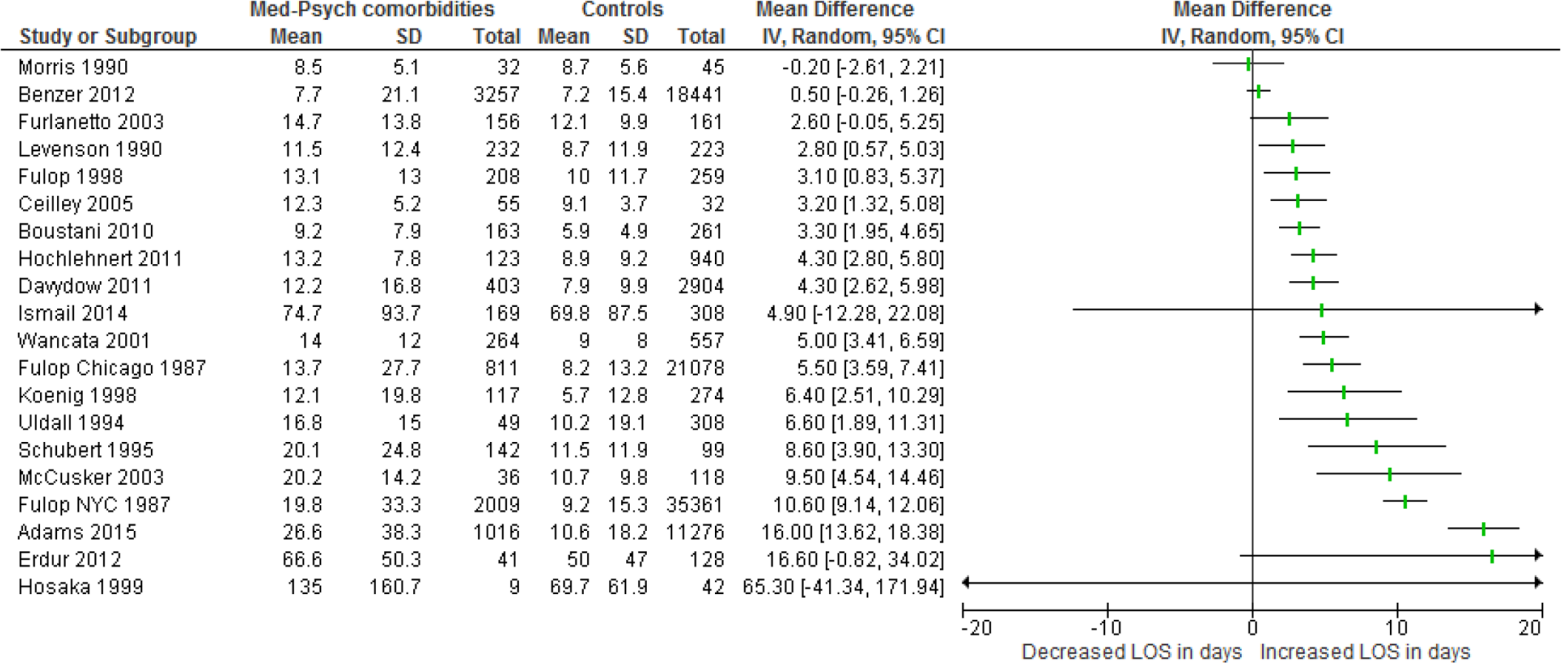
Relation of medical-psychiatric comorbidity and length of stay (LOS) (because the study of Fulop et al. [1987] included two separate samples in two hospitals, both outcomes are included in the analyses).

Nineteen (43%) of 42 studies provided data for meta-analysis “Fig 2”. The included studies appeared very heterogeneous: the range of the mean LOS in the comorbid group varied from 7.7–135.0 versus 7.2–69.8 in the control group. Because of this heterogeneity, the estimation of an overall pooled effect was not appropriate. Nevertheless, given that all results point in one direction, “Fig 2” shows that medical-psychiatric comorbidities are related to increased LOS.

#### Impact of medical-psychiatric comorbidities on medical costs

Table 3 shows the articles that described the impact of medical-psychiatric comorbidities on medical costs. Out of 12 studies, nine (75%) showed a significant relationship [8, 11, 29, 32, 36, 53, 58–60] and three (25%) showed a non-significant relationship [10, 27, 59].

**Fig 3:**
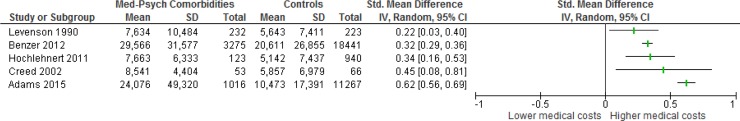
Relationship between medical-psychiatric comorbidity and medical costs.

The results of five (42%) studies were included in a meta-analysis “Fig 3”. These studies were very heterogenous and, therefore, an estimate of an overall pooled effect was not applicable. However, “Fig 3” reveals that medical-psychiatric comorbidities are related to increased medical costs.

#### Impact of medical-psychiatric comorbidities on rehospitalization

Table 3 shows the impact of medical-psychiatric comorbidities on rehospitalization. This relationship was described in ten (19%) studies [8, 9, 44, 45, 51, 61–65]; of these, nine (90%) revealed that medical-psychiatric comorbidities related to increased rehospitalizations. Seven (70%) studies found a significant increase [[8, 9, 44, 51, 61, 62, 64], two (20%) noted a non-significant increase [45, 65] and one (10%) found neither an increase nor decrease [63]. A meta-analysis was not executed since the data could not be used in a meta-analysis.

#### Impact of different subgroups on health-economic outcomes

In the reviewed studies, two subgroups–depression and delirium–appeared to be extensively studied and were suitable for further subgroup analysis.

#### Impact of comorbid depression and delirium on LOS

The relationship between comorbid depression and LOS was examined in 16 out of 43 (37%) studies; of these, 11 studies (69%) showed a significant relationship [23, 25, 31, 33, 35, 36, 40, 47, 49, 50, 54]; two studies (13%) showed a non-significant relationship [38, 51], two (13%) no effect [22, 26] and one (6%) study demonstrated a non-significant relationship between shorter LOS and comorbid depression [34].

**Fig 4:**
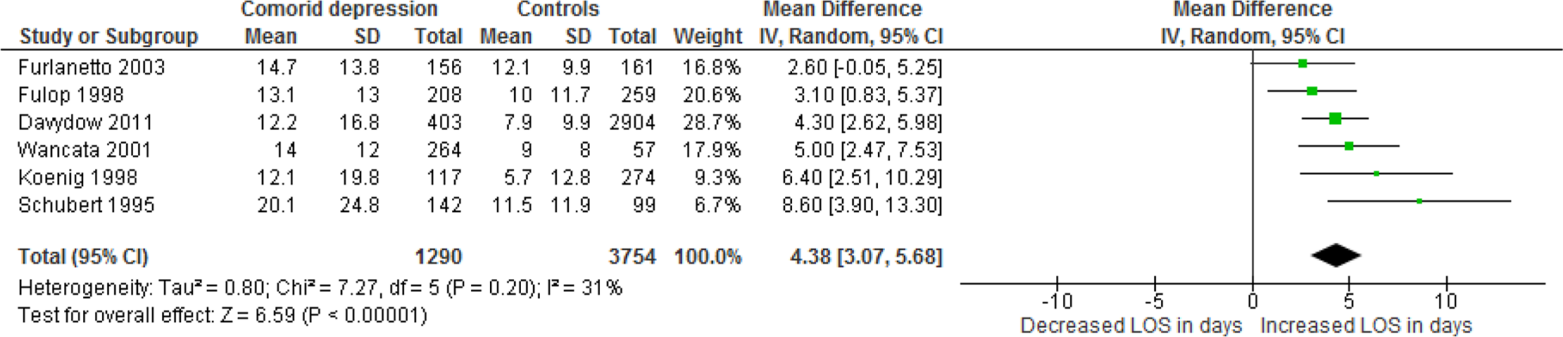
Meta-analysis in the subgroup depression examining the impact on length of stay (LOS).

Six studies provided suitable data for meta-analysis; the overall pooled mean difference in “Fig 4” showed that patients with comorbid depression had an LOS that was 4.38 days longer than patients without comorbid depression (mean LOS: 4.38 days; 95% CI: 3.07 to 5.68 days). The weighted average mean LOS of the depressed group was 13.8 days as opposed to 10.5 days for the non-depressed group.

Another extensively studied subgroup consisted of patients with comorbid delirium. Seven (16%) articles found a relationship between comorbid delirious patients and increased LOS compared to patients without comorbid delirium; five studies (71%) showed a significant relationship [32, 37, 42, 45, 52] and two studies (29%) a non-significant relationship [41, 51]. The data in these articles were not sufficient to perform a meta-analysis.

#### Impact of comorbid depression on medical costs

The relationship between comorbid depression and medical costs was examined in six (46%) studies; of these, five (92%) showed significantly higher medical costs in comorbid depressed patients compared to patients without comorbid depression [29, 36, 53, 58, 60]. One (8%) study indicated a non-significant relationship [32]. A meta-analysis was not performed as only two articles provided limited data.

#### Impact of comorbid depression on rehospitalization

The relationship between medical-psychiatric comorbidities and rehospitalization was, again, extensively examined in the subgroup with comorbid depression compared to patients without comorbid depression. This was described explicitly in four (40%) reviewed articles. Two (50%) studies found that the odds ratio for rehospitalization was significantly higher in patients with comorbid depression [51, 64]. Another study (25%) showed that patients with comorbid depression and a history of prior hospitalizations within six months were three times more likely to be rehospitalized within 90 days [61]. One (25%) study did not find a significant increase of the odds ratio for rehospitalization in patients with comorbid depression [63]. Meta-analysis for this subgroup was not possible because only one article provided useful data.

## Discussion

To our knowledge this is the first review that examined the relationship between medical-psychiatric comorbidity and health-economic outcomes. Our analysis shows that hospital inpatients having medical-psychiatric comorbidities have a longer LOS, higher medical costs and more rehospitalizations. No randomized trials were found, however, and only one included study reached the highest standardized NOS quality score. The pooled-effect measures were often not appropriate to interpret since the available studies were very heterogeneous [19]. Nevertheless, the (standardized) mean differences of all meta-analyses indicate that medical-psychiatric comorbidity is indeed related to increased health-economic outcomes. Moreover, the subgroup of depressed patients shows an increased mean LOS of 4.38 days compared to patients without depression: this outcome was moderately heterogeneous (I^2^ = 31%) and was therefore considered appropriate.

### Policy and clinical implications

Our systematic literature review elucidates the importance of medical-psychiatric comorbidities on health-economic outcomes. It is, consequently, disappointing to find that the quality of the included studies was mostly limited and that the heterogeneity of study samples was huge. Future studies on quality improvement strategies should therefore examine the impact of medical-psychiatric comorbidities on health-economic outcomes. This will help care providers and policy makers to organize care for patients with medical-psychiatric comorbidity in the most efficient way.

Our literature review suggests that the depressed subgroup of medical-psychiatric comorbid patients has stronger relationships with health-economics outcomes in comparison to non-depressed patients. For the first time in literature, we have established that, on average, hospital inpatients with comorbid depression stay in the hospital 4.38 days longer than non-depressed patients. In the Netherlands, the costs of a medical inpatient day range from €435 to €575 [66]. Based on the results of our review, the average medical costs in this country for patients with comorbid depression are increased to €1905-€2520 compared to patients without comorbidity. Researchers, care providers, policy makers and health insurers might use these outcomes for future research and healthcare policy making to improve cost-effective care for this subgroup. Furthermore, future prospective and randomized research should examine the impact of specific subgroups on health-economic outcomes more thoroughly to help hospitals improve cost-effective care for different subgroups with medical-psychiatric comorbidity.

### Limitations

Although a thorough and extensive electronic search was performed on 6163 titles, this systematic literature review had several limitations.

First, the included studies were highly heterogenic regarding the patient population, type of hospital, country and year; thus, the results were difficult to compare, which reduced the possibility of investigating a pooled effect. Therefore, the magnitude of the impact on health-economic outcomes remains uncertain.

Ideally, patients with medical-psychiatric comorbidity were compared to studies that examined the medical and psychiatric illness separately. In this way, it might be possible to examine whether the effect of different combinations of medical and psychiatric illness was additive or multiplicative. For reasons of feasibility, our search strategy was narrowed to the impact of medical-psychiatric comorbidity on inpatients (as opposed to inpatients without psychiatric comorbidity) on health-economic outcomes. It was anticipated that including all possible combinations of general medical and psychiatric illnesses in the search would lead to an unmanageable number of included papers.

Second, in the past decades, the average LOS in hospitals has been largely reduced, which made it harder to compare studies over time. Publication date was not an exclusion criterion; hence, some studies were published more than two decades ago. As such, the results in these studies could reflect hospital-care patterns that have since changed.

Next, the search in the literature list led to the inclusion of eight extra titles. These titles were not found in the extensive search strategy since they described a specific disorder and the focus of the search was on medical-psychiatric comorbidity in general. While developing the electronic search strategy, it was explicitly decided to not include specific disorders since there was no clear cut-off point to determine when to stop including specific disorders in the search terms. Furthermore, if all disorders that exist in the literature were included in the search, the results of the literature search would be too broad.

Subsequently, the studies that examined medical costs had some methodological variation in terms of sampling and reporting those costs. Some studies examined the impact of total costs (inpatient, outpatient, pharmacy) and some only the in-hospital costs. Nevertheless, the evidence of almost all studies concerning the relationship between medical costs and medical-psychiatric comorbidities pointed in the same direction. Consequently, it was suggested that comorbidity had an increasing effect on medical costs.

Moreover, the quality of the reviewed studies was variable and only one study received the full nine stars on the NOS. [17] Thus, the strength of the evidence differed among the three health-economic outcomes. Most studies examined the impact of medical-psychiatric comorbidities on LOS. As 95% of these studies showed an increased LOS, this relationship with medical-psychiatric comorbidities seemed the most reliable.

Another limitation of our study was that only one author rated the included articles. However, since almost all individual articles found results that pointed in the same direction, the risk of bias in the studies did not seem to impact the overall findings of this review. Additionally, there is a possible publication bias since only peer-reviewed articles were included. A study protocol was not developed in advance of this systematic review; it would therefore be challenging to note deviations and to assess if the outcomes of the review are reported according to the original study plan.

Finally, two subgroups of patients (comorbid depression and delirium) were comprehensively researched and therefore the results of studies that described these subgroups were used in the meta-analysis. This analysis was not predetermined but carried out post hoc since these groups appeared to be the most extensively studied in the included papers. Since the search was not focused on these subgroups, some literature that describes the impact of depression and a somatic comorbidity on health-economic outcomes may have been overlooked. Nevertheless, future researchers can use the results of this research to examine the impact of subgroups with a specific psychiatric disorder and a medical comorbidity.

Despite these limitations, the main conclusions of this review remain valid and clearly indicate, firstly, that general hospital inpatients with medical-psychiatric comorbidities showed longer LOS, higher medical costs and more rehospitalizations and, secondly, that the subgroup of depressed patients showed an increased mean LOS of 4.38 days compared to patients without depression.

## Conclusion

This systematic literature review found a relationship between medical-psychiatric comorbidities and health-economic outcomes (LOS, medical costs and rehospitalizations). The meta-analysis for the subgroup depression showed an increased LOS (on average 4.38 days longer inpatient stay) compared to patients without depression. These results demonstrate that, on average, the medical costs for this subgroup are between €1905 and €2550 higher than patients without a comorbid depression. Policy makers might use these results to improve cost-effective care for this subgroup. Based on our results, we suggest that future research should examine the impact of several subgroups with medical-psychiatric comorbidity on health-economic outcomes more thoroughly.

## Supporting information

S1 Table PRISMA checklist. (DOC)

S2 Table Final includes. (XLSX)

S3 Table Data extraction file. (XLSX)

S4 Table NOS grading risk of bias. (XLSX)

S5 Table Full search strategy. (DOCX)
