# Supplementary material for: Health-economic outcomes in hospital patients with medical-psychiatric comorbidity: A systematic review and meta-analysis
Source: PLoS One. 2018 Mar 13;13(3):e0194029. doi: 10.1371/journal.pone.0194029 (PMC5849295; doi:10.1371/journal.pone.0194029)
Supplement: S5 Table — (DOCX) [file pone.0194029.s005.docx]

## Attachment

## Search terms

**Embase.com**

(psychosomatics/exp OR (((somatic* OR physical* OR medical* OR medicine) NEAR/6 (psychiatr* OR mental* OR cognit*)) OR psychosomatic*):ab,ti) AND (comorbidity/exp OR 'cluster analysis'/exp OR (comorbid* OR cluster* OR ((multi* OR poly OR co) NEAR/3 (morbid* OR patholog*)) OR (co NEXT/1 exist*) OR coexist* OR (mixed NEAR/3 disorder*)):ab,ti) AND ('hospital patient'/exp OR hospitalization/exp OR outpatient/exp OR 'outpatient care'/exp OR 'ambulatory care'/exp OR 'outpatient department'/exp OR hospital/de OR 'general hospital'/de OR 'hospital admission'/exp OR 'hospital care'/exp OR 'university hospital'/exp OR 'hospital discharge'/exp OR 'hospital department'/de OR ward/de OR (hospital* OR inpatient* OR outpatient* OR ward* OR ambulator*):ab,ti) NOT ([Conference Abstract]/lim OR [Letter]/lim OR [Note]/lim OR [Conference Paper]/lim OR [Editorial]/lim) AND [english]/lim

**Medline (OvidSP)**

(Psychosomatic Medicine/ OR (((somatic* OR physical* OR medical* OR medicine) ADJ6 (psychiatr* OR mental* OR cognit*)) OR psychosomatic*).ab,ti.) AND (comorbidity/ OR cluster analysis/ OR (comorbid* OR cluster* OR ((multi* OR poly OR co) ADJ3 (morbid* OR patholog*)) OR (co ADJ exist*) OR coexist* OR (mixed ADJ3 disorder*)).ab,ti.) AND (inpatients/ OR exp hospitalization/ OR outpatients/ OR Ambulatory Care/ OR hospitals/ OR Hospitals, General/ OR Hospitals, University/ OR Hospital Departments/ OR (hospital* OR inpatient* OR outpatient* OR ward*).ab,ti.) NOT (letter OR news OR comment OR editorial OR congresses OR abstracts).pt. AND english.la.

**PsycINFO (OvidSP)**

(Psychosomatic Medicine/ OR (((somatic* OR physical* OR medical* OR medicine) ADJ6 (psychiatr* OR mental* OR cognit*)) OR psychosomatic*).ab,ti.) AND (comorbidity/ OR cluster analysis/ OR (comorbid* OR cluster* OR ((multi* OR poly OR co) ADJ3 (morbid* OR patholog*)) OR (co ADJ exist*) OR coexist* OR (mixed ADJ3 disorder*)).ab,ti.) AND (Hospitalized Patients/ OR hospitalization/ OR outpatients/ OR Outpatient Treatment/ OR hospitals/ OR (hospital* OR inpatient* OR outpatient* OR ward*).ab,ti.) NOT (letter OR news OR comment OR editorial OR congresses OR abstracts).pt. AND english.la.

**Cochrane**

((((somatic* OR physical* OR medical* OR medicine) NEAR/6 (psychiatr* OR mental* OR cognit*)) OR psychosomatic*):ab,ti) AND ((comorbid* OR cluster* OR ((multi* OR poly OR co) NEAR/3 (morbid* OR patholog*)) OR (co NEXT/1 exist*) OR coexist* OR (mixed NEAR/3 disorder*)):ab,ti) AND ((hospital* OR inpatient* OR outpatient* OR ward* OR ambulator*):ab,ti)

**Web-of-science**

TS=(((((somatic* OR physical* OR medical* OR medicine) NEAR/6 (psychiatr* OR mental* OR cognit*)) OR psychosomatic*)) AND ((comorbid* OR cluster* OR ((multi* OR poly OR co) NEAR/3 (morbid* OR patholog*)) OR (co NEAR/1 exist*) OR coexist* OR (mixed NEAR/3 disorder*))) AND ((hospital* OR inpatient* OR outpatient* OR ward* OR ambulator*))) AND DT=(Article) AND LA=(English)

**Google scholar**

"somatic|physical|medical psychiatric|mental" comorbidity|clustering|"co morbidity|existance" hospital|hospitalization|hospitalisation|outpatient|inpatient
